# MR1 blockade drives differential impact on integrative signatures based on circuits of circulating immune cells and soluble mediators in visceral leishmaniasis

**DOI:** 10.3389/fimmu.2024.1373498

**Published:** 2024-08-13

**Authors:** Luana Oliveira Borges-Fernandes, Marcela de Lima Moreira, Victória Hellena Silva Pereira, Marcelo Antônio Pascoal-Xavier, Ágata Lopes Ribeiro, Ismael Artur da Costa-Rocha, Ludmila Rosa Lopes, Guilherme Telles Cristo Moreira, Márcio Sobreira da Silva Araújo, Andréa Teixeira-Carvalho, Joaquim Pedro Brito-de-Sousa, Andrea Lucchesi de Carvalho, Maria Vitória Assumpção Mourão, Flávia Alves Campos, Marineide Borges, Mariângela Carneiro, Moriya Tsuji, Olindo Assis Martins-Filho, Jordana Grazziela Alves Coelho-dos-Reis, Vanessa Peruhype-Magalhães

**Affiliations:** ^1^ René Rachou Institute, Oswaldo Cruz Foundation (FIOCRUZ-MINAS), Belo Horizonte, Minas Gerais, Brazil; ^2^ School of Medicine, Federal University of Minas Gerais, Belo Horizonte, Minas Gerais, Brazil; ^3^ Basic and Applied Virology Laboratory, Department of Microbiology, Institute for Biological Sciences, Federal University of Minas Gerais, Belo Horizonte, Minas Gerais, Brazil; ^4^ João Paulo II Children’s Hospital, Fundação Hospitalar do Estado de Minas Gerais, Belo Horizonte, Minas Gerais, Brazil; ^5^ Parasitology Department, Universidade Federal de Minas Gerais, Belo Horizonte, Minas Gerais, Brazil; ^6^ Aaron Diamond AIDS Research Center, Columbia University Vagelos College of Physicians and Surgeons, New York, NY, United States; ^7^ Division of Infectious Disease, Department of Medicine, Columbia University Vagelos College of Physicians and Surgeons, New York, NY, United States

**Keywords:** MR1 blockade, *L. infantum*, phagocytes, soluble mediators, integrative circuits, cytokines, nitric oxide, internalization

## Abstract

**Introduction:**

Visceral leishmaniasis (VL) is an important tropical and neglected disease and represents a serious global health problem. The initial interaction between the phagocytes and the parasite is crucial to determine the pathogen’s capacity to initiate infection and it shapes the subsequent immune response that will develop. While type-1 T-cells induce IL-6, IL-1β, TNF-α, and IL-12 production by monocytes/macrophages to fight the infection, type-2 T-cells are associated with a regulatory phenotype (IL-10 and TGF-β) and successful infection establishment. Recently, our group demonstrated the role of an important Th1/Th17 T-cell population, the mucosal-associated invariant T (MAIT) cells, in VL. MAIT cells can respond to *L. infantum* by producing TNF-α and IFN-γ upon MR1-dependent activation.

**Objective and methods:**

Here, we describe the impact of the MR1-blockage on *L. infantum* internalization on the functional profile of circulating neutrophils and monocytes as well as the impact of the MR1-blockage on the soluble mediator signatures of *in vitro* whole blood cultures.

**Results:**

Overall, our data showed that VL patients presents higher percentage of activated neutrophils than asymptomatic and non-infected controls. In addition, MR1 blockade led to lower TNF-α and TGF-β production by non-activated neutrophils from asymptomatic individuals. Moreover, TNF-α and IL-10 production by monocytes was higher in VL patients. In the analysis of soluble mediators produced *in vitro*, MR1-blockade induced a decrease of IFN-γ and an increase of IL-10, IL-27 and IL-33 in the cell cultures of AS group, a cytokine pattern associated with type 2 deleterious response.

**Discussion and conclusion:**

These data corroborate the hypothesis that MR1-restricted responses are associated to a protective role during *Leishmania* infection.

## Introduction

Visceral leishmaniasis (VL) is one of the seven most important tropical and neglected diseases that presents a wide spectrum of clinical manifestations and can be fatal in more than 95% of cases if not treated (1). Patients with classical VL may present anemia, leukopenia, hepatosplenomegaly, and intermittent fever, while severe VL is associated with bleeding, splenomegaly, edema, weakness, jaundice, bacterial pneumonia, and sepsis (2, 3). Indeed, severe VL is related to exacerbation of the systemic inflammatory cytokine response, especially IL-6, IL-1β, TNF-α and IFN-γ. This enhanced cytokine response is associated with macrophage activation syndrome (MAS) along with increased production of localized regulatory cytokines, notably IL-10, IL-27 and TGF-β (4–7).

Neutrophils take a crucial role as the initial cellular responders to the infections caused by *Leishmania donovani* (8) and *Leishmania infantum* (9), shaping the course of the infection by regulating parasite control. These cells play diverse functions in this regard, including phagocytosis, secretion of enzymes and antimicrobial proteins, oxidative bursts, and the formation of Neutrophil Extracellular Trap (NETs) (10, 11). Furthermore, neutrophils contribute actively to the modulation of adaptive immune responses by inducing the secretion of several cytokines such as TNF-α, IL-12, and IFN-γ (12–15). *Leishmania* primarily targets monocytes/macrophages as its host cells, and the initial interaction between the parasite and the target cell is crucial to determine the pathogen’s capacity to initiate the infection and shapes the subsequent immune response (16). In response to cytokines produced by Type 1 T-cells (Th1), monocytes/macrophages exhibit a pro-inflammatory phenotype, which is characterized by increased production of IL-6, IL-1β, TNF-α, and IL-12, as well as by important antimicrobial agents such as NO (Nitric Oxide) and ROS (Reactive Oxygen Species). Conversely, when stimulated by Type 2 T-cells (Th2) cytokines, monocytes/macrophages undertake a regulatory phenotype marked by elevated levels of IL-10 and TGF-β, which are deleterious to the host and are associated with successful establishment of the parasites in the host (17–21).

In addition to Th1 and Th2 immune responses, the importance of cytokines produced by Th17/22 axis in controlling *L. infantum* and *L. donovani* infections has been demonstrated (22). For instance, Th17 and γδ T-cells were found to be important sources of IL-17A in the liver and spleen of mice infected with *L. donovani* (23). An important subset of unconventional innate-like T-cells are the mucosal-associated invariant T (MAIT) cells. These cells have unique circulation patterns and a significant impact in the host’s immune response against the infection, characterized as cytotoxic (24–26) and effector memory (27) profile. MAIT cells possess an invariant TCR α-chain consisting of the semi-variable region (TRAV) 1-2 and joining region (TRAJ) 33 that recognize antigens limited to the non-classical antigen-presenting molecule, the MHC class I-related protein 1 (MR1), which is expressed by diverse cell types as antigen-presenting cells (APCs) (28, 29). Although, MAIT cells can be found in various tissues, they are found at high frequencies in organs that play crucial role in the pathology of VL, such as liver, intestine, lymphoid organs, and spleen (30–33). Regarding this context, recently our group have demonstrated that, upon MR1-dependent activation, MAIT cells can respond to *L. infantum* by producing TNF-α and IFN-γ (34). However, it is not known how the blockade of these MR1-interactive pathways may impact the response of phagocytes during VL.

Here, we describe the impact of the MR1 blockade on *L. infantum* internalization and in the functional profile of circulating neutrophils and monocytes as well as the impact of the MR1 blockade in the soluble mediator signatures of *in vitro* whole blood cultures.

## Material and methods

### Study population

Clinical samples from children (age 2 – 9) with classical visceral leishmaniasis (LV) were obtained before (LV-BT) and after (LV-AT) treatment at the João Paulo II Children’s Hospital, Belo Horizonte, Minas Gerais, Brazil. All participants had diagnosis confirmed by positive serology using the IT Leish^®^ assay (Bio-Rad Laboratories, Hercules, CA, USA). Whole blood samples were collected before (VL-BT group) and after (VL-AT group) 20-40 days of treatment with meglumine antimoniate (used in 64% of the cases) or liposomal amphotericin B (used in 29% of the cases). Asymptomatic patients presenting positive tests for ELISA (rk39) or whole blood real-time PCR were also included in the study. In addition, endemic groups, recruited from metropolitan region of Belo Horizonte, were included, comprising the asymptomatic (AS group, n=18) and non-infected healthy children (NI group, n=15) groups. Written informed consent was obtained from parents and/or legal guardians of the participants before inclusion in the study. The research adhered to the guidelines outlined in the Brazilian resolution 466/2012 established by the National Commission on Research Ethics (CONEP). This study protocol was submitted and approved by the Ethics Committee of the René Rachou Institute, IRR/FIOCRUZ-MINAS (protocol #782.042) and the João Paulo II Children’s Hospital (protocol #860.893).

### *L. infantum* labeling

Promastigotes of *L. infantum* (MHOM/BR/1974/PP75) were obtained from *in vitro* axenic culture in biphasic blood-agar medium (Novy-McNeal-Nicolle plus Liver Infusion Triptose supplemented with 10% of fetal bovine serum (FBS) over rabbit blood agar). Cultures were maintained at 26°C in a Biochemical Oxygen Demand incubator. A metacyclic-enriched promastigote suspension was collected at the stationary growth phase (7-days of culture) and submitted to differential low-speed centrifugation at 100 x g for 10 minutes, at 18°C, to spin down parasites clumps and debris. After 20 minutes incubation at 26°C, single cell parasites suspension was recovered in the supernatant and transferred to a 50 mL polypropylene tube. The parasites were washed twice with phosphate-buffered saline (PBS) supplemented with 10% FBS by high-speed centrifugation at 900 x g for 10 minutes at 18°C. The pellet was resuspended in 1 mL of PBS with 10% FBS, the parasites counted in a Neubauer chamber on optical microscopy and adjusted to obtain a promastigote suspension at 1.0 x 10^8^ parasites/mL. An aliquot of parasite suspension (2 mL) was incubated with 100 μL of Alexa Fluor 647 solution (Invitrogen, Thermo Fisher Scientific – MA, USA) at 3.2 g/mL final concentration, for 30 minutes at 37°C in the dark. Following incubation, fluorescent-labeled parasites were submitted to three washing steps with PBS supplemented with 10% FBS at 900 x g for 10 minutes at 18°C and the promastigote suspension adjusted to a final concentration of 1.0 x 10^8^ fluorescent parasites/mL in a final volume of 1 mL. The labeling quality control of fluorescent-labeled parasites was carried out on a BD LRS Fortessa Flow Cytometer (BD Biosciences, San Jose, CA, USA), considering the mean fluorescence intensity (MFI) target between 10^3^ and 10^4^ monitored on single parameter histograms.

### Whole blood cultures

Heparinized whole blood samples were centrifuged at 660 x g for 10 minutes at 18°C for plasma removal. The cells were resuspended in RPMI 1640 medium (GIBCO – Grand Island, NY, USA), gently homogenized and centrifugated at 660 x g for 10 minutes at 18°C. After supernatant removal, the cellular concentration was adjusted to 1.0 x 10^7^ leukocytes/mL. To assess the parasite internalization and intracytoplasmic cytokine profile, three different *in vitro* whole blood cultures were performed, referred as: Control Culture (CC), *L. infantum* (1:2 parasite:cells ratio) and *L. infantum* (1:2 parasite:cells ratio) + α-MR1 antibody (10μg/mL; clone 26.5). Briefly, after 1 hour of incubation, at 37°C in a 5% CO_2_ humidified atmosphere, 10μg/mL of Brefeldin A (BFA - Sigma, MO, USA) was added to each whole blood culture to evaluate the intracytoplasmic cytokine profile. After 4 hours of incubation, 20mM of EDTA (Sigma, MO, USA) was added in the cultures, following assessment of intracytoplasmic cytokine profile by flow cytometry. Cultures without BFA were performed for assessing immune soluble factors secreted in the supernatant.

### Intracytoplasmic cytokine profile

For the monocyte and neutrophil phagocyte surface marker analysis, 200μL of the cell suspension of each culture condition, was incubated with anti-HLA-DR-FITC (clone: Tu39), anti-CD16-AF700 (clone: 3G8) and anti-CD14-V450 (clone: MφP9) monoclonal antibodies (BD Biosciences, San Jose, CA, USA). After 20 minutes incubation in the dark at room temperature, erythrocytes were lysed (FACS Lysing Solution – BD, San Jose, CA, EUA) and leucocytes were permeabilizated with 200μL of PBS-P (PBS solution with 0.5% bovine serum albumin, 0,5% saponin and 0.1% sodium azide). After permeabilization, 200μL of PBS-W (PBS solution with 0.5% bovine serum albumin and 0.1% sodium azide) was added to the cultures and 40μL of the cell suspensions were transferred to 96 wells U-bottom plates (Sarstedt, Inc – Newton) containing the phycoerythrin (PE)-labeled monoclonal antibodies: anti-IFN-γ (clone: 4S.B3), anti-TNF-α (clone: MAb11), anti-IL-10 (clone: JES3-9D7), anti-IL-12 (clone: C11.5) and anti-TGF-β (clone: TB21), all purchased from BD Biosciences (San Jose, CA, USA). The plates were incubated for 30 minutes in the dark, at room temperature. After incubation, the cells were washed with PBS-P followed by PBS-W. After centrifugation at 600 x g, for 7 minutes at 18°C, cells were resuspended with 200μL of PBS and the cell suspensions were maintained at 4°C for 15 minutes. A total of 3,000 events within the CD14^+^ monocyte gate was acquired using BD LRS Fortessa Flow Cytometer (BD Biosciences, San Jose, CA, USA). The phenotypic profile, internalization rate of *L. infantum*, and intracellular cytokine production by phagocytes were analyzed using FlowJo (version 10.1, Tree Star Inc. Ashland, OR, USA), at the Flow Cytometry Platform facility at FIOCRUZ-MINAS.

### Nitric Oxide (NO) production by whole blood phagocytes

Aiming at assessing the intracellular nitric oxide (NO) production in peripheral blood phagocytes, a flow cytometric assay was performed according to Schachnik and colleagues (35). Briefly, the assay is based on 4,5-diaminofluorescein diacetate probe (DAF-2DA) that after being deacetylated by intracellular esterases to DAF-2, it reacts with NO to yield fluorescent DAF-2T (triazolofluorescein) compound, which shows a green fluorescence detected by the flow cytometer. Briefly, the cultures (control, *L. infantum* and *L. infantum* + α-MR1) were conducted using leukocytes aliquots (1.0 x 10^7^ cells/mL), which were incubated for 1 hour with the DAF-2DA, at 37°C in a 5%CO_2_ humidified atmosphere. Lipopolysaccharide from *Escherichia coli* (LPS, 10mg/mL; Sigma, MO, USA) was employed as iNOS inducer (positive control culture). For specificity assays, Aminoguanidine (AG, 10 mM; Sigma, MO, USA), an iNOS inhibitor, was used. Following, the samples were incubated for 20 minutes in the dark at room temperature in the presence of anti-HLA-DR-PE (clone: G46-6), anti-CD16-AF700 (clone: 3G8) and anti-CD14-V450 (clone: MφP9) monoclonal antibodies (BD Biosciences, San Jose, CA, USA). Following incubation, the samples were washed with PBS at 600 x g, for 7 minutes at 18°C and a total of 3,000 events/CD14^+^ monocyte gate, were acquired using BD LRS Fortessa Flow Cytometer (BD Biosciences, San Jose, CA, USA). The data were analyzed by FlowJo (version 10.1, Tree Star Inc. Ashland, OR, USA), at the Flow Cytometry Platform facility at FIOCRUZ-MINAS.

### Assessment of soluble mediators

The supernatants from Control (CC), *L. infantum* and *L. infantum* + α-MR1 cultures were collected, and the levels of soluble mediators were measured by Luminex immunoassay. The quantification of chemokines, pro-inflammatory cytokines, Th17/22 axis cytokines, regulatory cytokines, and growth factors was performed using the “Milliplex MAP Kit – Th17” system (Merck Millipore, Burlington, MA, EUA) and the “The Bio-Plex Pro Human Cytokine 27-Plex” system (BioRad Laboratories, Hercules, CA, USA), following the manufacturer’s instruction. The results were expressed in pg/mL according to standard curves inserted on each experimental batch. The data was analyzed by Bio-plex 200 System (BioRad Laboratories, CA, USA) at Flow Cytometry Platform facility at FIOCRUZ-MINAS.

### Statistical analysis

Data analysis was performed using GraphPad Prism v.9.1.1 software (San Diego, CA, USA). Considering the non-parametric distribution of the data, comparative analyzes were performed using Mann-Whitney test or Kruskal-Walli’s test, followed by Dunn’s post-test. Significant differences at p<0.05 are represented by the letters “a” “b” “c” and “d” as compared to NI, AS, VL-BT and VL-AT groups, respectively. Heatmap constructs were assembled using Microsoft Excel (Albuquerque, NM, USA) to illustrate the functional profiles of neutrophils and monocytes in NI, AS, VL-BT and VL-AT groups. Lollipop charts show the relationship between a numeric and a categoric variable. Here, lollipop charts were plotted to represent the proportion (%) of samples with soluble mediator index (*L.i*/CC and *L.i* + α-MR1/CC) above the global median cut-off in NI, AS, VL-BT and VL-AT groups. Correlation analysis was employed to build integrative networks of soluble mediators. Pearson and Spearman correlation tests were used to obtain the significant “r” scores at p<0.05 and the Cytoscape software platform (https://cytoscape.org) was employed to construct the integrative networks. The integrative networks were organized using cluster layouts comprising five categories of parameters including: chemokines, pro-inflammatory, regulatory, and Th17/Th22 cytokines along with growth factors. The node sizes are proportional to the number of correlations between biomarkers. Descriptive analysis was represented by orbital charts summarizing the number of correlations for each study group. Changes in biomarker connectivity were estimated as *L.i.* + α-MR1/*L.i.* ratio to identify soluble mediators with increased (>2x) or decreased (<0.5x) connectivity.

## Results

### Impact of MR1 blockade on *L. infantum* internalization and nitric oxide production by circulating neutrophils and monocytes

**Figure 1:**
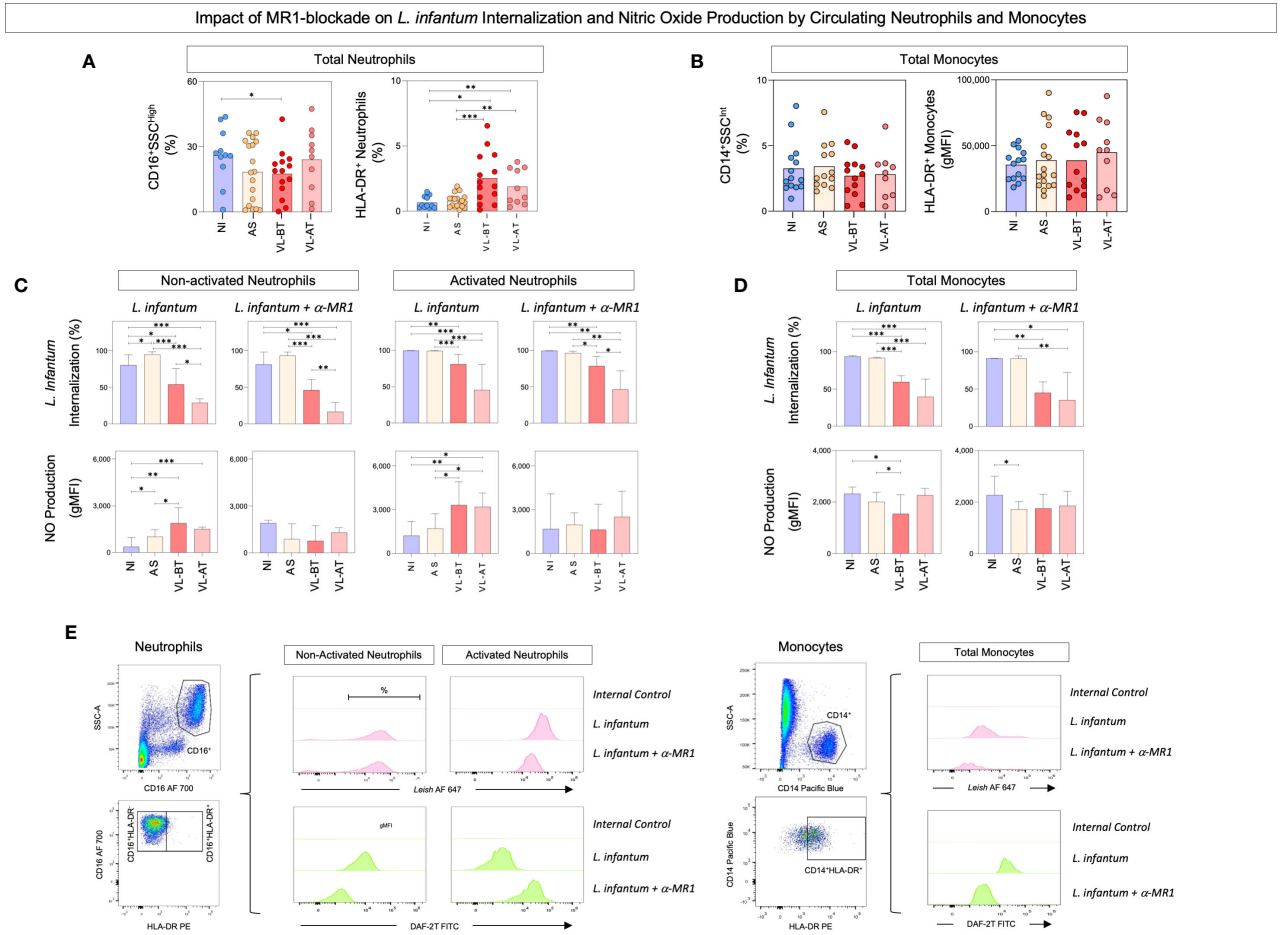
Impact of MR1 blockade on *L. infantum* internalization and nitric oxide production by circulating neutrophils and monocytes. **(A)** The frequencies (%) of circulating neutrophil and **(B)** total monocytes were quantified in whole blood samples from non-infected endemic controls (NI), asymptomatic individuals (AS) along with visceral leishmaniasis patients before (VL-BT) and after treatment (VL-AT) by flow cytometry, as described in Material and Methods. Data are shown as scattering distribution of individual values over bar charts representing the median values. Significant differences amongst groups were considered at p<0.05 and indicated by connecting lines. **(C)** The percentage of *L. infantum* internalization and NO production by activated and non-activated neutrophils and **(D)** total monocytes were determined upon in vitro whole blood cultures referred as: *L. infantum* and *L. infantum* + α-MR1, as described in Material and Methods. The results are shown in bar charts representing the median (IQR) values for the percentage of *L. infantum* internalization and the geometric mean of Mean Fluorescence Intensity (gMFI) for NO production. Significant differences amongst groups were indicated by connecting lines, and the asterisks represent the significancy levels: *p < 0.05, **p < 0.01, and ***p < 0.001. **(E)** Representative flow cytometric charts using pseudocolor density plots illustrate the strategies used to selected neutrophils subsets (Total – CD16^+^SSC^High^, non-activated - HLA-DR^–^CD16^+^ and activated - HLA-DR^+^CD16^+^) and total monocytes (CD14^+^SSC^Int^; CD14^+^DR^+^) and histograms to quantify the percentage of *L. infantum* internalization and NO production.

To evaluate the impact of MR1 blockade on the phagocytic capacity and oxidative potential of circulating neutrophils and monocytes, whole blood samples from non-infected (NI) and asymptomatic (AS) individuals, as well as patients with visceral leishmaniasis before (LV-BT) and after (LV-AT) treatment were stimulated with live *L. infantum* promastigotes in the absence/presence of α-MR1 mAb. The rates of *L. infantum* internalization and nitric oxide production were assessed by flow cytometry and the results are presented in **Figure 1**. Data analysis demonstrated that regardless of the lower frequency of total neutrophils (CD16^+^SSC^high^) in comparison to NI, VL-BT presented higher percentage of activated neutrophils (HLA-DR^+^) than NI and AS. Likewise, VL-AT also exhibited more activated neutrophils as compared to NI and AS (**Figure 1A**). No differences were observed for the frequency or activation of total monocytes (CD14^+^SSC^int^) amongst groups (**Figure 1B**). Additionally, the ability of neutrophils to internalize *L. infantum* in patients before and after treatment (VL-BT and VL-AT) was impaired as compared to NI individuals, regardless of MR1 blockade. Conversely, AS individuals exhibited higher *Leishmania* internalization by non-activated neutrophils in *L. infantum* culture in comparison to all other groups (**Figure 1C** – top panels). Neutrophils from VL-BT and VL-AT patients, and non-activated neutrophils from AS individuals produced higher levels of NO in *L. infantum*-stimulated culture as compared to NI donors. Interestingly, the MR1 blockade inhibited the NO production by neutrophils in those three groups, reaching similar levels as observed for NI, both in non-activated and activated cells (**Figure 1C** – bottom panels).

Lower rates of *Leishmania* internalization were observed in monocytes from VL-BT and VL-AT groups as compared to NI and AS, in both culture conditions (**Figure 1D** – top panels). Monocytes from VL-BT produced less NO as compared to NI and AS in the *L. infantum* stimulated culture, and the addition of MR1 blockade to the cultures lead to lower NO production by monocytes from AS compared to NI (**Figure 1D** – bottom panels).

Representative flow cytometric charts containing the gating strategies used to selected neutrophils and monocytes subsets as well as the approaches used to quantify the *L. infantum* internalization and NO production are illustrated on **Figure 1E**.

### Impact of MR1 blockade on the functional profile of circulating neutrophils and monocytes

**Figure 2:**
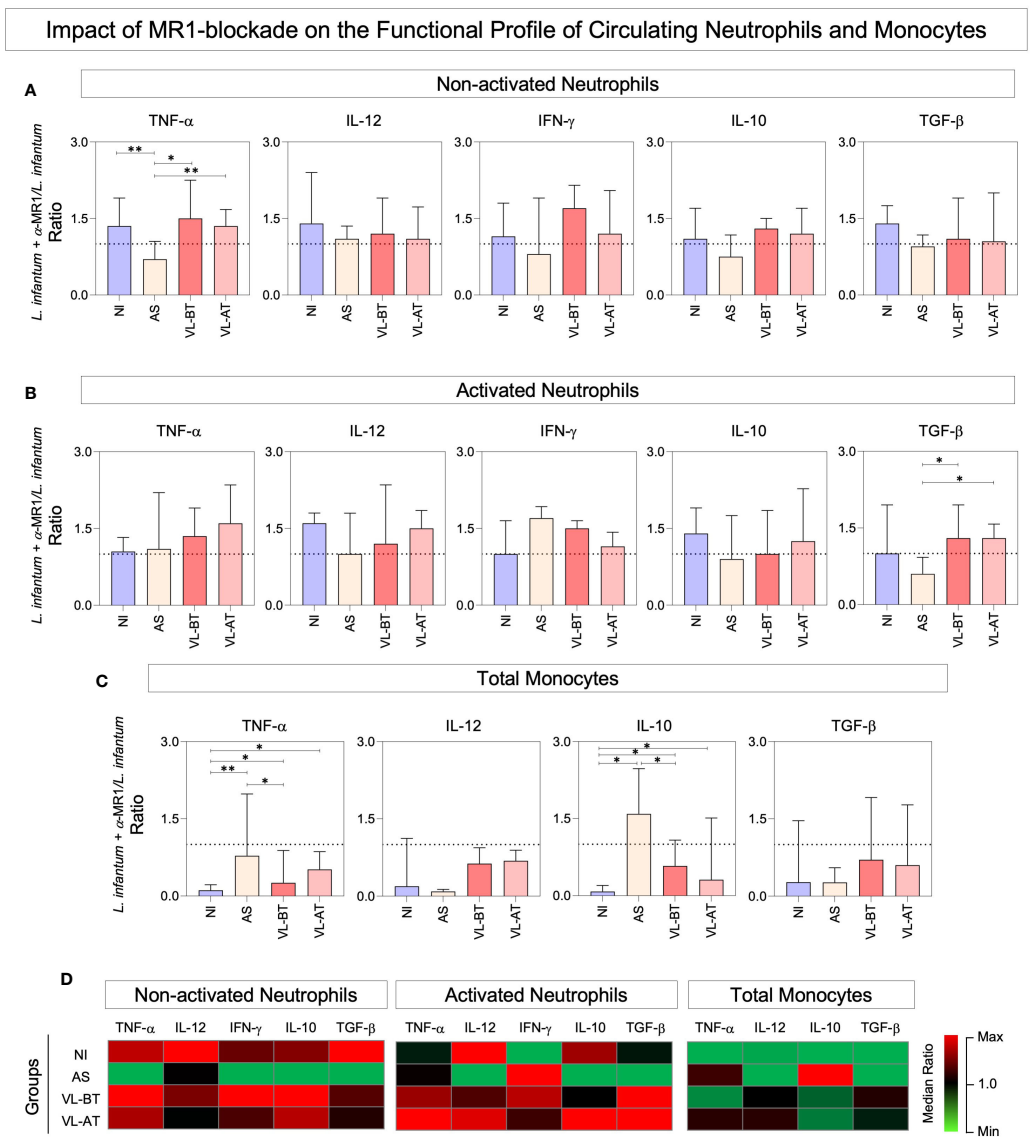
Impact of MR1 blockade on the functional profile of circulating neutrophils and monocytes. **(A)** The frequencies (%) of non-activated neutrophils and **(B)** activated neutrophils expressing (TNF^+^, IL-12^+^, IFN-γ^+^, IL-10^+^ and TGF-β^+^) and **(C)** total monocytes expressing (TNF^+^, IL-12^+^, IL-10^+^ and TGF-β^+^) were quantified in whole blood samples from non-infected endemic controls (NI), asymptomatic individuals (AS) along with visceral leishmaniasis patients before (VL-BT) and after treatment (VL-AT) by intracytoplasmic flow cytometry, as described in Material and Methods. Data are shown in bar charts representing the median (IQR) values of *L. infantum* + α-MR1/*L. infantum* ratio. Significant differences amongst groups were indicated by connecting lines, and the asterisks represent the significancy levels: *p < 0.05, and **p < 0.01. **(D)** Heatmap constructs illustrate the functional profiles neutrophils and monocytes in NI, AS, VL-BT and VL-AT groups.

The functional profile of circulating neutrophils and monocytes was assessed in whole blood cultures upon stimuli with live *L. infantum* promastigotes in the absence/presence of α-MR1 blocking mAb. The results are expressed as ratio of *L. infantum* + α-MR1/*L. infantum* cultures and are presented in **Figure 2**. Data analysis demonstrated that the MR1 blockade led to lower expression of TNF-α by non-activated neutrophils from AS group in comparison to all other groups (**Figure 2A**). Moreover, MR1-blockage reduced the TGF-β production by activated neutrophils from AS when compared to all other groups (**Figure 2B**). Additionally, TNF-α and IL-10 production by monocytes was higher in VL-BT and VL-AT as compared to NI and even higher in AS in comparison to NI and VL-BT (**Figure 2C**). Heatmap constructs further corroborate these findings illustrating that, overall, the presence of MR1 blockade impaired the functional response of neutrophils in NI, VL-BT and VL-AT, while it improved the response of monocytes, particularly in AS individuals (**Figure 2D**).

### Impact of *in vitro* MR1 blockade on soluble mediator signatures of whole blood cultures

**Figure 3:**
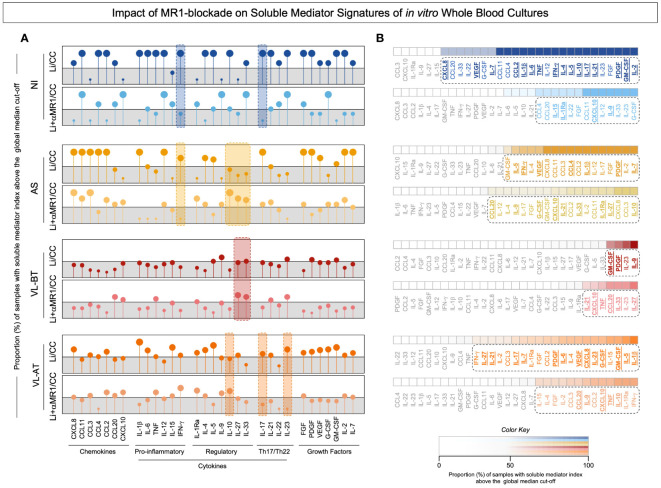
Impact of MR1 blockade on soluble mediator signatures of in vitro whole blood cultures. **(A)** The soluble mediators chemokine (CXCL8, CCL11, CCL3, CCL4, CCL2, CCL20, CXCL10), pro-inflammatory cytokines (IL-1β, IL-6, TNF, IL-12, IL-15, IFN-γ), regulatory cytokines (IL-1Ra, IL-4, IL-5, IL-9, IL-10, IL-27, IL-33), Th17/Th22 axis cytokines (IL-17, IL-21, IL-22, IL-23) and growth factors (FGF, PDGF, VEGF, G-CSF, GM-CSF, IL-2, IL-7) were measured in the supernatant of *in vitro* whole blood cultures from non-infected endemic controls (NI), asymptomatic individuals (AS) along with visceral leishmaniasis patients before (VL-BT) and after treatment (VL-AT) by Luminex Bio-plex platform, as described in Material and Methods. **(A)** The data are shown in Lollipop charts representing the proportion (%) of samples with soluble mediator index (*L.i*/CC and *L.i* + α-MR1/CC) above the global median cut-off (grey zone). Qualitative features associated with MR1 blockade on each group was underscored by color dashed rectangles. **(B)** Heatmap constructs illustrate the ascendant soluble mediator signatures. The biomarkers with proportion above the 50^th^ percentile were highlighted by dashed rectangles and the soluble mediators increased in *L.i*/CC and *L.i* + α-MR1/CC culture condition on each group underscored by bold underline format.

To investigate the impact of the MR1 blockade on the expression of soluble mediators regulating anti-*Leishmania* immunity, we established signatures of chemokines, cytokines, and growth factors present in the supernatant of our *in vitro* cultures stimulated with live *L. infantum* promastigotes in the absence/presence of α-MR1 mAb. The results expressed as the proportion (%) of samples with soluble mediator index (*L. infantum*/CC and *L. infantum* + α-MR1/CC) above the global median cut-off and are presented in **Figure 3**. Data demonstrated that MR1 blockade induced distinct signatures in each clinical group. From the quantitative point of view, MR1 blockade reduced the set of soluble mediators with an increased index in NI and VL-AT. Conversely, the MR1 blockade increased the number of soluble mediators with increased index in AS and VL-BT (**Figure 3A**). Qualitative analysis indicated that MR1 blockade was associated with a decrease of IFN-γ and IL-17A in NI, while in AS, it was associated with a decrease of IFN-γ and an increase of IL-10, IL-27 and IL-33. Increase of IL-27 and IL-33 upon MR1 blockade were reported for VL-BT, whereas reduction of IL-17 and IL-23 with upregulation of IL-10 was identified in VL-AT (**Figure 3A**, dashed color rectangles). Ascendant soluble mediator signatures further pointed out the selected soluble mediators increased in the absence or presence of MR1 blockade (**Figure 3B**). The analysis of common and selective soluble mediators revealed distinct sets of attributes associated with each clinical group, however, increased CXCL10 secretion was universally attributed to MR1 blockade (**Figure 3B**).

### Impact of MR1 blockade on soluble mediator integrative networks of *in vitro* whole blood cultures

**Figure 4:**
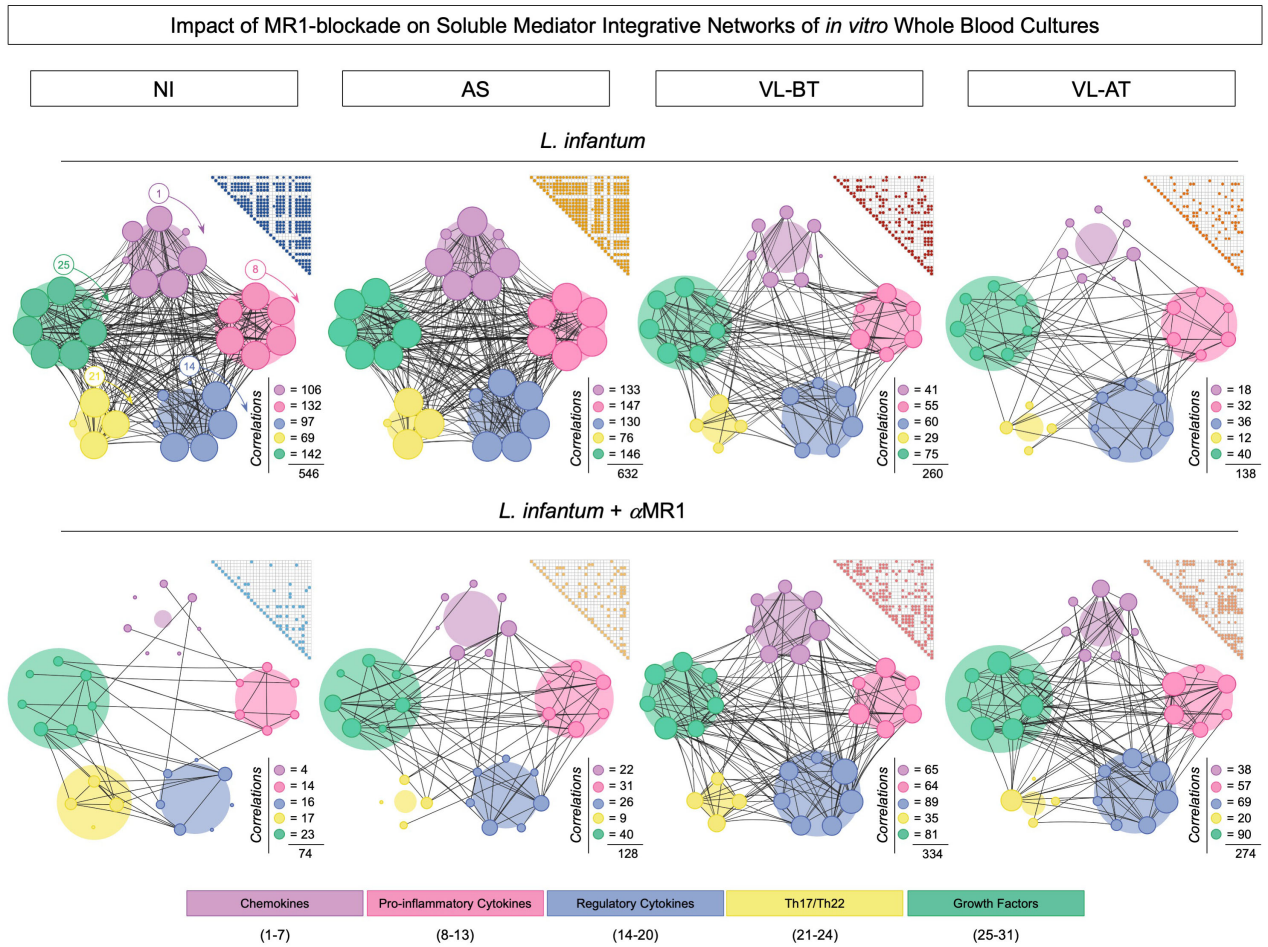
Impact of MR1 blockade on soluble mediator integrative networks of in vitro whole blood cultures. Comprehensive correlation matrices and networks of chemokines (CXCL8, CCL11, CCL3, CCL4, CCL2, CCL20, CXCL10), pro-inflammatory cytokines (IL-1β, IL-6, TNF, IL-12, IL-15, IFN-γ), regulatory cytokines (IL-1Ra, IL-4, IL-5, IL-9, IL-10, IL-27, IL-33), Th17/Th22 axis cytokines (IL-17, IL-21, IL-22, IL-23) and growth factors (FGF, PDGF, VEGF, G-CSF, GM-CSF, IL-2, IL-7) were assembled based on the significant (p<0.05) “r” correlation scores obtained for *in vitro* whole blood cultures (*L. infantum* and *L. infantum* + α-MR1) from non-infected endemic controls (NI) and asymptomatic individuals (AS) along with visceral leishmaniasis patients before (VL-BT) and after treatment (VL-AT), as described in Material and Methods. Triangle matrices were assembled to compile the “r” correlation scores for each group. The integrative networks were constructed using cluster layouts comprising five categories of parameters including: chemokines (1-7), pro-inflammatory (8-13), regulatory (14-20) and Th17/Th22 cytokines (21-24) along with growth factors (25-31). The node sizes are proportional to the number of correlations between biomarkers. The number of correlations involving each category as well as the total correlation number are provided in the figure. Circular backgrounds underscore the proportional contribution of each category of parameters to the overall connectivity.

Integrative networks of soluble mediators were built to further characterize the impact of the MR1 blockade in the *in vitro* whole blood culture. The results are presented in **Figure 4**. Data analysis demonstrated that while NI (n=546) and AS (n=632) groups exhibited a higher number of correlations upon *L. infantum* stimuli, lower connectivity between soluble mediators was observed for VL-BT (n=260) and VL-AT (n=138). Conversely, the connections between soluble mediators waned upon MR1 blockade in NI (n=74) and AS (n=128), whereas a slight increase in connectivity was identified for VL-BT (n=334) and VL-AT (n=274) (**Figure 4**).

**Figure 5:**
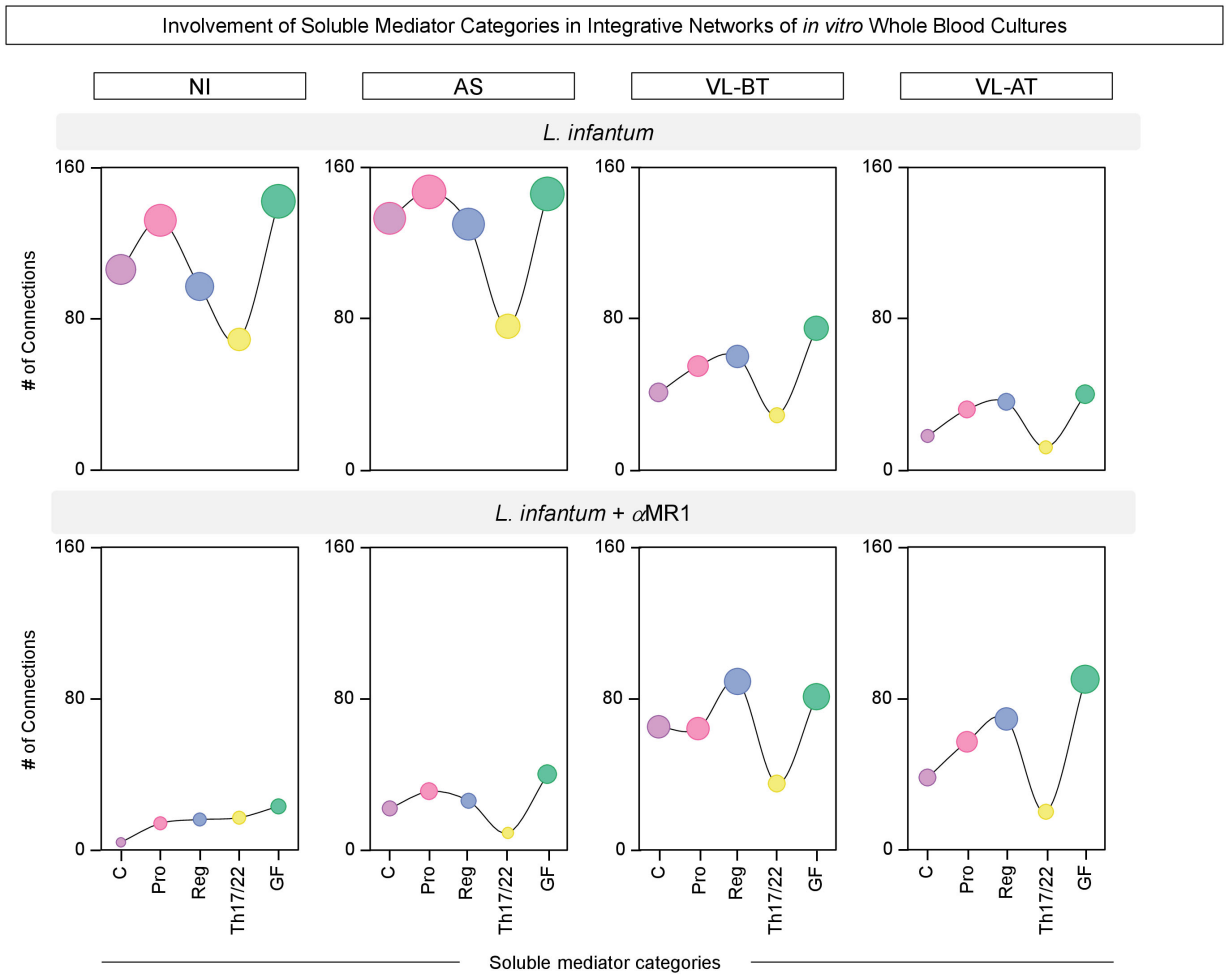
Involvement of soluble mediator categories in integrative networks of in vitro whole blood cultures. The number of correlations involving categories of soluble mediators, including: chemokines (C), pro-inflammatory (Pro), regulatory (Reg), Th17/Th22 axis (Th17/22) cytokines and growth factors (GF) was calculated from integrative networks of *in vitro* whole blood cultures (*L. infantum* and *L. infantum* + α-MR1) from non-infected endemic controls (NI) and asymptomatic individuals (AS) along with visceral leishmaniasis patients before (VL-BT) and after treatment (VL-AT). Data are shown as line charts with symbol sizes proportional to the number of correlations involving each category of soluble mediator.

To further characterize the distinct involvement of soluble mediators in the overall connectivity profile, we quantified the connections in each soluble mediator cluster C (chemokine), Pro (pro-inflammatory), Reg (regulatory), Th17/22 and GF (grow factor), for each clinical group (**Figure 5**). Data demonstrated that the depletion of connections observed in NI and AS upon MR1 blockade impacted all soluble mediator clusters, while in VL-BT and VL-AT, the increase in connectivity was more prominent in the Reg and GF clusters (**Figure 5**).

**Figure 6:**
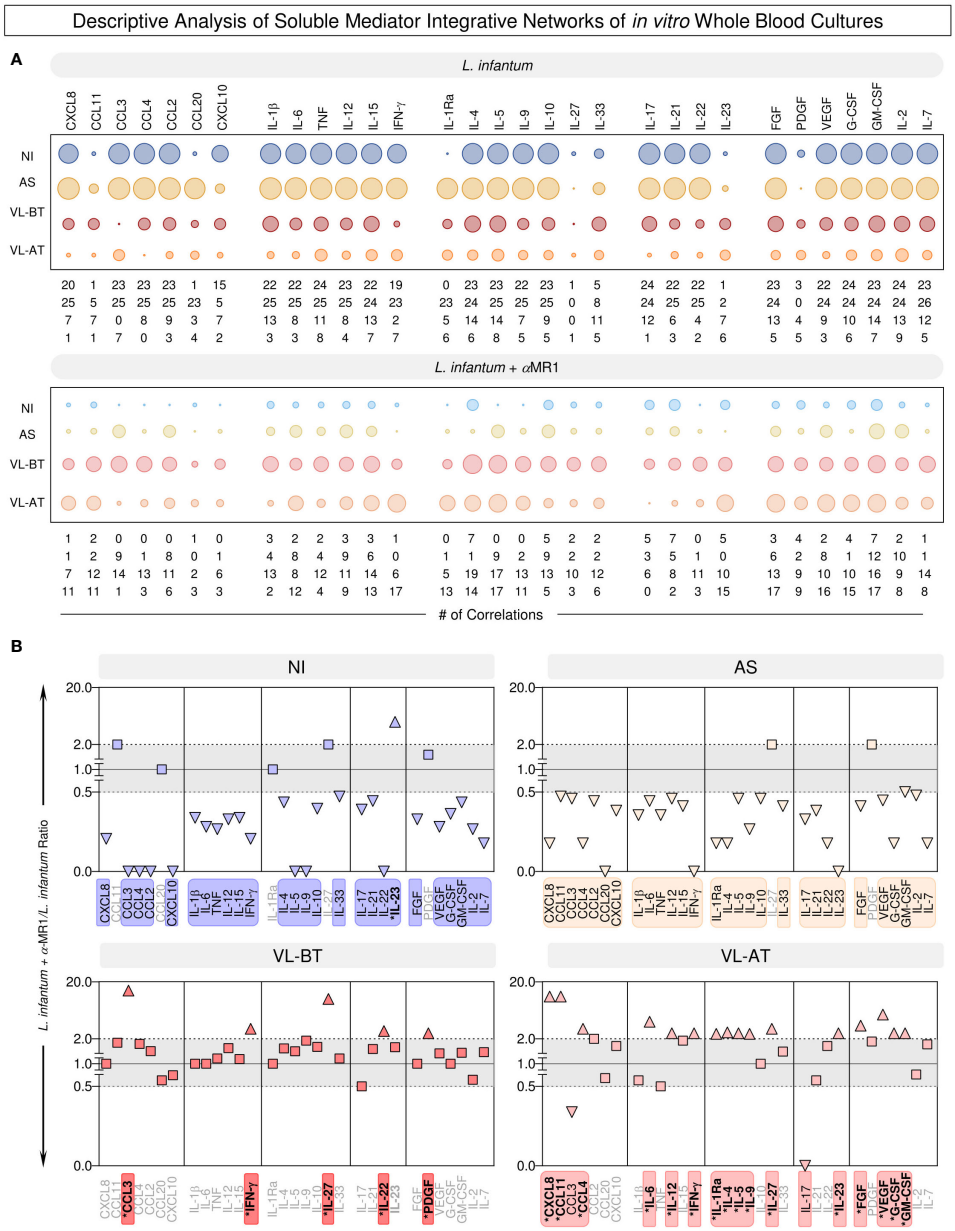
Descriptive analysis of soluble mediator integrative networks of in vitro whole blood cultures. **(A)** The number of correlations involving chemokines (CXCL8, CCL11, CCL3, CCL4, CCL2, CCL20, CXCL10), pro-inflammatory cytokines (IL-1β, IL-6, TNF, IL-12, IL-15, IFN-γ), regulatory cytokines (IL-1Ra, IL-4, IL-5, IL-9, IL-10, IL-27, IL-33), Th17/Th22 axis cytokines (IL-17, IL-21, IL-22, IL-23) and growth factors (FGF, PDGF, VEGF, G-CSF, GM-CSF, IL-2, IL-7) was assembled from integrative networks of *in vitro* whole blood cultures (*L. infantum* and *L. infantum* + α-MR1) from non-infected endemic controls (NI) and asymptomatic individuals (AS) along with visceral leishmaniasis patients before (VL-BT) and after treatment (VL-AT). Data are shown as orbital charts summarizing the number of correlations for each group. **(B)** Changes in biomarker connectivity upon MR1 blockade were estimated as *L. infantum* + α-MR1/*L. infantum* ratio to identify soluble mediators with increased (>2x) or decreased (<0.5x) connectivity. Color frames and backgrounds highlight the sets of soluble mediators with changes in connectivity. * Underscores the biomarkers with increased connectivity upon MR1 blockade. Squares represents the biomarkers that did not presented changes (0.5>x>2x) in connectivity and triangles represents the biomarkers that did present changes.

Aiming at further understanding the connectivity profile between soluble mediators, descriptive analysis of the integrative networks was performed to identify sets of soluble mediators with increased (>2x) or decreased (<0.5x) connectivity upon MR1 blockade. These results are presented in **Figure 6**. The results further evidenced the differential impact of MR1 blockade on the integrative networks across the distinct clinical groups. While MR1 blockade substantially reduced the connectivity throughout all sets of soluble mediators in NI and AS, minor increases were observed in VL-BT and VL-AT (**Figure 6A**). In NI and AS, the loss of connectivity in *L. infantum* + α-MR1/*L. infantum* ratio involved all sets of soluble mediators (**Figure 6B**). Conversely, a restricted set of soluble mediators (CCL3, IFN-γ, IL-27, IL-22 and PDGF) drove the increased connectivity observed in VL-BT. Of note, the impact of MR1 blockade in VL-AT was more robust and affected a larger number of soluble mediators (CXCL8, CCL11, CCL4, IL-6, IL-12, IFN-γ, IL-1Ra, IL-4, IL-5, IL-9, IL-27, IL-23, PFGF, VEGF, G-CSF and GM-CSF) (**Figure 6B**).

Additionally, analysis was carried out to further identify the impact of MR1 blockade on selective circuits within each clinical group (**Supplementary Figure 1**). Data demonstrated that changes in the pattern of intra-circuit connectivity upon MR1 blockade are specific for each clinical group (**Supplementary Figure 1A**). The NI group was characterized by loss of connectivity in chemokines and by the increase in Th17/22 and growth factors clusters. In AS, increase in connectivity was exclusively observed in the pro-inflammatory cytokine cluster. Both VL-BT and VL-AT presented a decrease in connectivity involving Th17/22 and an increase in connectivity of clusters containing growth factors. Particularly in VL-BT, the MR1 blockade led to a decrease of connectivity within the pro-inflammatory cluster (**Supplementary Figure 1B**). All in all, these results suggest important impact of MR1 blockade in cluster connectivity of soluble mediators of VL before and after treatment as well as, AS and endemic controls.

## Discussion

MR1 is a non-classical antigen-presenting molecule MHC class I-related. Unlike other antigen-presenting molecules (MHC-I and MHC-II), in the absence of infection, MR1 is hardly detectable on the surface of human cells. The MR1 molecule is retained in the endoplasmic reticulum, and it is readily upregulated in ligands presence (28, 29).

The importance of MR1 molecule and MR1-restricted cells has been described in a wide spectrum of infectious diseases caused by fungi, bacteria, and viruses (36–40). Notably, our research group previously revealed the involvement of MR1-restricted cells in a protozoan infection caused by Leishmania (34).

In this study, we describe the impact of the MR1 blockade on *L. infantum* internalization and on the functional profile of circulating neutrophils and monocytes as well as the impact of the MR1 blockade in the soluble mediator signatures *in vitro*. These analyses were carried out upon *in vitro* whole blood cultures referred as: *L. infantum* and *L. infantum* + α-MR1. Due to ethical restriction for blood samples collect from children, we were unable to conduct additional cultures, including: Cells + α-MR1, Cells + α-IL-12/Il-18, and *L. infantum* + α- IL-12/IL-18.

Numerous studies have characterized the phenotypic and functional changes in phagocytes, including both polymorphonuclear and mononuclear cells, in the context of *Leishmania* spp. and various other pathogens. These investigations have contributed significantly to our understanding about MHC-I and MHC-II antigen presentation (41). However, there is a notable lack of literature aiming at elucidating specific alterations occurring in phagocytes concerning antigen presentation via MR1 and the consequences of blocking this molecule within these cells. To our knowledge, the current study stands out as the pioneering effort to shed light on the impact of MR1 blockade on phagocytes upon *Leishmania* spp. infection.

Considering the Leishmania infection, neutrophils are the initial innate immune cells to be recruited to the infection site (8, 9, 42). Neutrophils exhibit various anti-parasitic mechanisms, such as the phagocytosis of parasites (10, 11). Previous studies have demonstrated that neutrophils from patients with VL can phagocyte a comparable number of *L. donovani* as neutrophils from healthy controls (43, 44). However, our analysis revealed a different outcome: both activated and non-activated neutrophils from VL patients exhibit reduced Leishmania internalization as compared to non-infected (NI) individuals, which could be related to differences in our *in vitro* method. The impaired rates of Leishmania internalization profiles are found even after treatment. In line with these observations, a secondary role of neutrophils has been suggested regarding the ability of those cells in facilitating Leishmania infection of monocytes when they undergo apoptosis (45, 46). Monocytes play a pivotal role in Leishmania infection as the primary host cells. The primary route of infection involves direct phagocytosis of the remaining parasites by these monocytes (47). In a study performed by Singh and colleagues (48), monocytes from patients with VL exhibited a reduced phagocytic ability as compared to those from endemic controls, corroborating with our findings. Monocytes from VL patients presented reduced phagocytic capacity when compared to monocytes from NI and AS individuals, before and after treatment. Furthermore, our investigation revealed that the MR1 blockade did not alter this phagocytic profile, highlighting the persistence of this impairment in VL patients. Previous findings have established a connection between Toll-Like Receptors (TLRs) and the internalization of Leishmania by neutrophils and monocytes (49, 50). In our study, we investigated whether the blockade of the MR1 molecule could modify the internalization pattern. However, our analysis demonstrated that the internalization pattern remained unaltered, indicating that MR1 does not exert a significant influence on this process.

Both neutrophils and monocytes produce NO as an anti-Leishmania mechanism. Our data showed increase in the NO production by neutrophils in VL patients as compared to NI group. Neutrophils have been shown to play a protective role during visceral leishmaniasis (8, 9, 51). In experimental models it has been showed that neutrophils are activated and produce TNF-α and NO in a TLR2-dependent manner, after *L. infantum* stimulation (51). In fact, neutrophils have an independent role in orchestrating the early cytokine/chemokine responses, and actively contribute to molding adaptive immune responses against the parasite (8, 16). Our data corroborate with these studies showing elevated frequency of neutrophils IL-12^+^, IFN-γ^+^, TNF-α^+^, IL-10^+^, and TGF-β^+^, especially in VL groups (**Figure 2D**).

Despite the relevance of elucidating the role of monocytes subsets (CD14^++^CD16^-^ and CD14^dim^CD16^+^) in Leishmania infection (52) in the present study, the functional profile of monocytes was evaluated using total CD14^+^, since it was not possible to accurately access the functional profile of monocytes subsets, mainly due to the low monocytes counts in peripheral blood from VL patients and the ethical restriction to collect large amount of samples from children. Several reports indicate that patients with VL exhibit lower levels of NO production by total monocytes in comparison to healthy controls (51, 52). Considering the relevance of characterizing the functional aspects of classical (CD14^++^CD16^-^) and non-classical (CD14^dim^CD16^++^) monocytes upon MR1-blockade, additional studies would contribute to bring about insightful and interesting findings to elucidate the role of monocytes subsets in Leishmania infection. Our results are in accordance with these observations.

Here, we showed a high NO production by total monocytes in NI and AS individuals as compared to VL patients. It has been described that monocyte exhibited distinct behaviors depending on the type of activation. Several studies have established that the development of Th1 responses, characterized by the production of cytokines such as TNF-α and IL-12, leads to the clearance of parasites and resistance to leishmaniasis. In contrast, a Th2 type response, marked by elevated levels of IL-10 and TGF-β, is associated with susceptibility to infection, disease progression, parasite replication and persistence (17, 19, 20, 53). Our findings indicate that the presence of the MR1 blockade leads to an increase in IL-10 levels in AS individuals. This result aligns with the observation that MR1 blockade contributes to reducing the IFN-γ levels and increasing the IL-10, IL-27 and IL-33 levels in the supernatant of *in vitro* cultures from the AS group. This scenario raises the possibility that the MR1 molecule may have a protective role in the context of VL, since high levels of IL-10 is a well-established feature of patients with classical VL (54). Furthermore, IL-27 has been recognized as a factor that promotes disease, by facilitating the differentiation and expansion of T cells that produce IL-10 *in vitro* (55, 56). The induction of IL-10 can occur through various pathways involving IL-27 (57). Recently, elevated IL-27 levels were associated to dysfunctional immune response to infectious diseases, potentially leading to significant tissue damage (58).

Further analysis of the supernatant compartment revealed that blocking MR1 increased the secretion of CXCL10, which was found as a common feature present across all the clinical groups. Previous studies have illustrated that parasite-infected Kupffer cells swiftly release CXCL10 to attract inflammatory monocytes and T-cells, thereby forming a granuloma that initiates anti-parasitic immune responses (59, 60). Moreover, CXCL10 has the potential to enhance protective immune responses during VL, by upregulating pro-inflammatory mediators, Th1-cell cytokines, and NO production *in vivo* (61). In a recent study, it was noted that CXCL10 could suppress IL-10^+^ Treg-cells within the spleen of mice infected with *L. infantum*, resulting in a reduction of the parasite load in the spleen of these mice (62). Conversely, excessive production of CXCL10 and CXCL9 has been associated with disease severity in VL, potentially leading to tissue damage due to uncontrolled inflammation (63, 64).

In addition to the well-recognized Th1 and Th2 immune responses, Th17 cells appear as a significant protective subset that contributes to controlling VL infection (65, 66). It has been demonstrated that IL-17A collaborates with IFN-γ to enhance NO production in infected macrophages (67). Besides, the cytokines associated with the Th17 axis strongly correlates with the asymptomatic VL. This protective effect is related to an increase in CXCL chemokines, which serve as potent chemoattractants for both neutrophils and Th1-cells, ultimately contributing to parasite clearance (66, 68). Terrazas and colleagues reported that Tγδ cells are a significant source of IL-17A in the spleen of VL patients (23). Tγδ cells are known to produce IL-17A in response to stimuli like dendritic cell-derived cytokines, such as IL-1β or IL-23, or direct interaction with pathogens (69). In addition, another significant “innate-like” T cell subset comprise MAIT cells, which is recognized for expressing high levels of IL-17A in a variety of infections and non-infectious diseases (70–72).

Despite our ability to extract a substantial amount of data from the provided samples, it is crucial to recognize the encountered limitations. The “VL-AT” group, evaluated within the initial 20-40 days after treatment, might not comprehensively depict the long-term dynamics of cytokine kinetics. Future studies are yet required in order to evaluate the immunological profile of patients after 90 and 180 days of treatment to expand our understanding on the impact of treatment on cytokine production.

In summary, our findings provide evidence of the impact of the MR1 blockade in the functional profile of phagocytes and the overall production of soluble mediators in the context of VL. Our results also support the hypothesis about the protective role of MR1 and MR1-restricted cells in asymptomatic VL individuals. Nevertheless, further investigations are warranted to address extensively and precisely the roles played by MR1 and MR1-restricted cells in protozoan infections, particularly in the Visceral Leishmaniasis.

## Data availability statement

The original contributions presented in the study are included in the article/**Supplementary Material**. Further inquiries can be directed to the corresponding author/s.

## Ethics statement

The studies involving humans were approved byComitê de Ética – Instituto René Rachou - Fiocruz/Minas Av. Augusto de Lima, 1715 – Barro Preto - Belo Horizonte (Cep: 30190-002) Telefone (31): 3349 7825 e-mail: cepcoord.minas@fiocruz.br. The studies were conducted in accordance with the local legislation and institutional requirements. Written informed consent for participation in this study was provided by the participants’ legal guardians/next of kin.

## Author contributions

LB-F: Conceptualization, Data curation, Formal analysis, Investigation, Methodology, Writing – original draft, Writing – review & editing. MD: Conceptualization, Data curation, Investigation, Methodology, Validation, Writing – original draft. VP: Formal analysis, Investigation, Methodology, Writing – original draft, Writing – review & editing. MP-X: Conceptualization, Formal analysis, Writing – review & editing. ÁL: Formal analysis, Writing – review & editing. IC-R: Formal analysis, Methodology, Writing – original draft, Writing – review & editing. LL: Formal analysis, Investigation, Methodology, Writing – review & editing. GM: Formal analysis, Investigation, Methodology, Writing – review & editing. MA: Formal analysis, Investigation, Methodology, Writing – review & editing. AT-C: Conceptualization, Formal analysis, Investigation, Methodology, Writing – review & editing. JPB: Formal analysis, Visualization, Writing – original draft, Writing – review & editing. AC: Methodology, Writing – review & editing. MM: Methodology, Writing – review & editing. FC: Methodology, Writing – review & editing. MB: Methodology, Writing – review & editing. MC: Methodology, Writing – review & editing. MT: Conceptualization, Formal analysis, Writing – review & editing. OM-F: Conceptualization, Formal analysis, Writing – original draft, Writing – review & editing. JC-R: Conceptualization, Data curation, Formal analysis, Funding acquisition, Investigation, Methodology, Project administration, Resources, Software, Supervision, Validation, Visualization, Writing – original draft, Writing – review & editing. VP-M: Conceptualization, Data curation, Formal analysis, Funding acquisition, Investigation, Methodology, Project administration, Resources, Software, Supervision, Validation, Visualization, Writing – original draft, Writing – review & editing.
