# Supplementary material for: MR1 blockade drives differential impact on integrative signatures based on circuits of circulating immune cells and soluble mediators in visceral leishmaniasis
Source: Front Immunol. 2024 Aug 13;15:1373498. doi: 10.3389/fimmu.2024.1373498 (PMC11347828; doi:10.3389/fimmu.2024.1373498)
Supplement: Supplementary file 1 [file DataSheet_1.pdf]

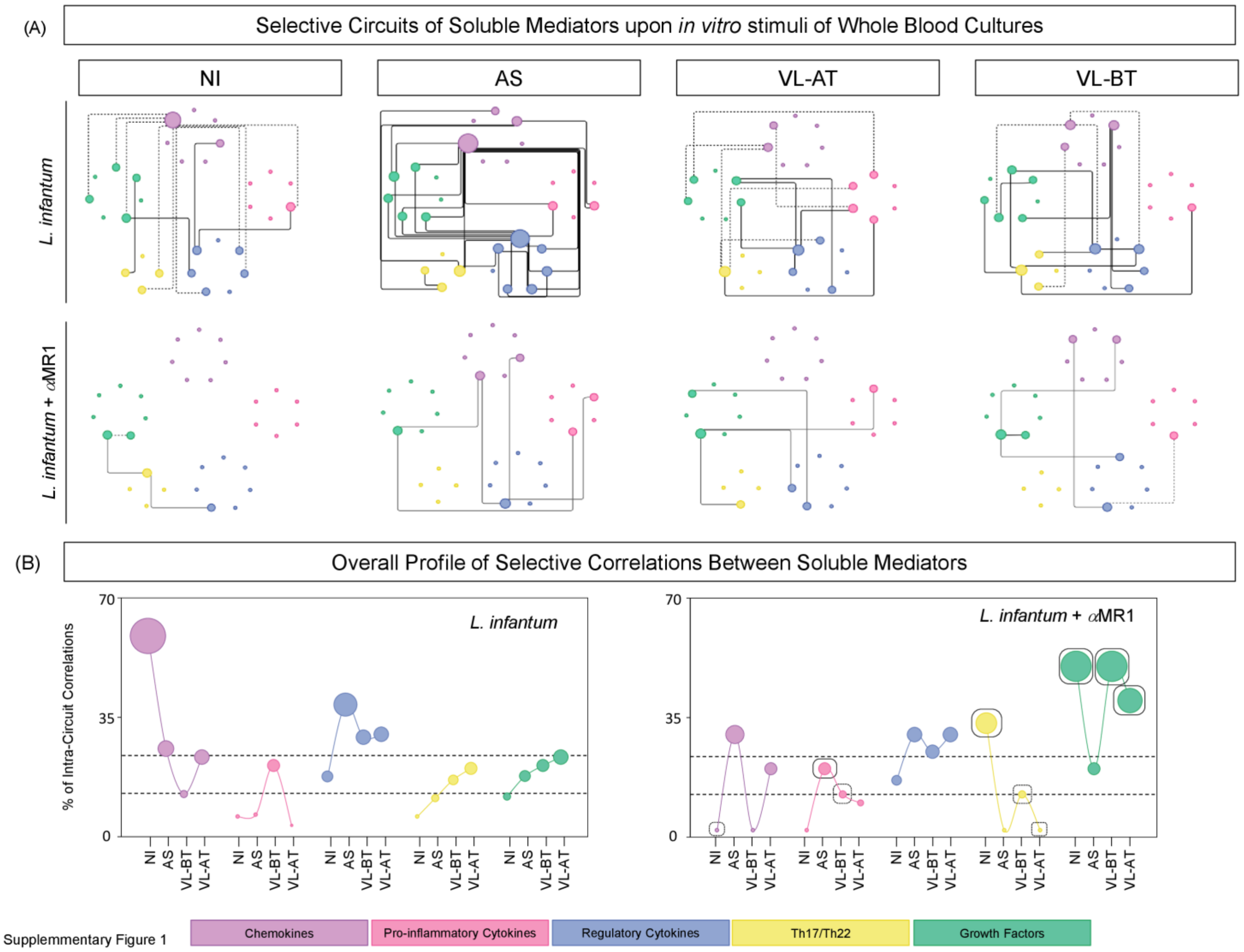

Supplementary Figure 1. Selective circuits of soluble mediators upon in vitro *stimuli of whole blood cultures.* **(A)** Comprehensive networks were assembled to display the selective correlations observed for *L. infantum* or *L. infantum* + a-MR1 *in vitro* whole blood cultures from non-infected endemic controls (NI) and asymptomatic individuals (AS) along with visceral leishmaniasis patients before (VL-BT) and after treatment (VL-AT), as described in Material and Methods. The selective circuits were built using cluster layouts comprising five categories of parameters including: chemokines (purple), pro-inflammatory (pink), regulatory (blue) and Th17/Th22 cytokines (yellow) along with growth factors (green). The node sizes are proportional to the number of correlations between biomarkers. Continuous or dashed connecting lines represent positive or negative correlations, respectively. The percentage of intra-circuit correlations involving each category are provided in the figure. **(B)** Line charts illustrate the percentage of intra-circuit correlations of selective correlations between soluble mediators amongst clinical groups. The symbol sizes are proportional to the percentage of intra-circuit correlations. Shifts in the percentage of intra-circuit correlations upon MR1-blockage are underscored by continuous or dashed rectangles to identify increase or decrease, respectively.
